# *COL4A4* variant recently identified: lessons learned in variant interpretation—a case report

**DOI:** 10.1186/s12882-022-02866-9

**Published:** 2022-07-16

**Authors:** Jenelle Cocorpus, Megan M Hager, Corinne Benchimol, Vanesa Bijol, Fadi Salem, Sumit Punj, Laura Castellanos, Pamela Singer, Christine B Sethna, Abby Basalely

**Affiliations:** 1 Pediatric Nephrology, Cohen Children’s Medical Center of New York, 269-01 76th Avenue, New Hyde Park, NY 11040 USA; 2 Natera, San Carlos, CA USA; 3 Pediatric Nephrology, Mount Sinai, New York, NY USA; 4 Renal Pathology, Northwell Health, Lake Success, NY USA; 5 Pathology, Molecular and Cell Based Medicine, Mount Sinai, New York, NY USA

**Keywords:** Case report, Alport syndrome, COL4A4, Genetic testing, Variant interpretations

## Abstract

**Background:**

Alport syndrome is a hereditary kidney disease characterized by hematuria and proteinuria. Although there have been reports of autosomal dominant *COL4A4* variants, this is likely an underdiagnosed condition. Improved access to affordable genetic testing has increased the diagnosis of Alport syndrome. As genetic testing becomes ubiquitous, it is imperative that clinical nephrologists understand the benefits and challenges associated with clinical genetic testing.

**Case Presentation:**

We present a family of Mexican descent with a heterozygous *COL4A4* variant (c.5007delC, ClinVar accession numbers: SCV001580980.2, SCV001993731.1) not previously discussed in detail in the literature. The proband received a biopsy diagnosis suggestive of Fabry disease 18 years after she first developed hematuria and progressed to chronic kidney disease stage III. One year later, the proband was provisionally diagnosed with Alport syndrome after a variant of uncertain significance in the *COL4A4* gene was identified following targeted family variant testing of her daughter. Upon review of the medical histories of the proband’s children and niece, all but one had the same variant. Of the four with the variant, three display clinical symptoms of hematuria, and/or proteinuria. The youngest of the four, only months old, has yet to exhibit clinical symptoms. Despite these findings there was a considerable delay in synthesizing this data, as patients were tested in different commercial genetic testing laboratories. Subsequently, understanding this family’s inheritance pattern, family history, and clinical symptoms, as well as the location of the *COL4A4* variant resulted in the upgrade of the variant’s classification. Although the classification of this variant varied among different clinical genetic testing laboratories, the consensus was that this variant is likely pathogenic.

**Conclusions:**

This *COL4A4* variant (c.5007delC) not yet discussed in detail in the literature is associated with Alport syndrome. The inheritance pattern is suggestive of autosomal dominant inheritance. This report highlights the intricacies of variant interpretation and classification, the siloed nature of commercial genetic testing laboratories, and the importance of a thorough family history for proper variant interpretation. Additionally, the cases demonstrate the varied clinical presentations of Alport syndrome and suggest the utility of early screening, diagnosis, monitoring, and treatment.

## Background

Advancements in genetic testing have made it widely accessible for diagnosis and treatment of patients with kidney disease [1, 2]. Alport syndrome (AS) is one of the most common inherited kidney diseases [3]. AS is characterized by hematuria and proteinuria with varying degrees of additional clinical symptoms. The genes associated with AS are *COL4A3, COL4A4,* and *COL4A5*, which encode for the α3, α4, or α5 chains of collagen IV that are responsible for formation of the glomerular basement membrane (GBM) and other basement membranes [2, 4, 5]. While kidney biopsy was initially the primary method for the diagnosis of AS, genetic testing is a more definitive and noninvasive method of diagnosis [6].

While genetic testing is crucial for confirmation of the AS diagnosis, its use poses several challenges [7, 8]. One is the phenotypic spectrum exhibited by individuals heterozygous for *COL4A4* or *COL4A3* variants. Although a majority of *COL4A4* or *COL4A3* pathogenic variants have been associated with autosomal recessive Alport syndrome (ARAS), there are also variants associated with autosomal dominant Alport syndrome (ADAS) [9, 10]. Patients with ADAS can have symptoms ranging from benign, non-progressive microscopic hematuria to kidney failure. Kidney failure is less common, but if these patients progress to kidney failure, it occurs later in life. They may also present with alternate thinning and thickening of the GBM, but without lamellations that are commonly seen in patients with ARAS and X-linked AS [11, 12]. Growing evidence also suggests that there are more pathogenic variants in *COL4A4* and *COL4A3* than previously reported [13, 14]. Thus, there is likely an underdiagnosis of individuals with a singular *COL4A4* or *COL4A3* variant. A second challenge is the siloed nature of genetic testing. Despite the recommended American College of Medical Genetics and Genomics (ACMG) classification guidelines, internal classification systems vary at different laboratories [15]. Although sometimes data is shared on resources like ClinVar, unpublished laboratory data that is not shared can result in different interpretations [16–19]. This makes it feasible that a single family, with the same phenotypic presentation, that had genetic testing performed at separate laboratories had different interpretations of the same variant. Identifying novel variants associated with disease, sharing genetic test findings, detailing the history of patients more thoroughly, and classifying variants uniformly among laboratories could greatly enhance the ability of clinicians to inform, advise, and treat their patients.

Therefore, we present a case study of a family of Mexican descent with a *COL4A4* variant that was classified by consensus as likely pathogenic among different clinical genetic testing laboratories, has not yet been discussed in detail in the literature, and highlights the above points.

## Case Presentation

**Fig. 1:**
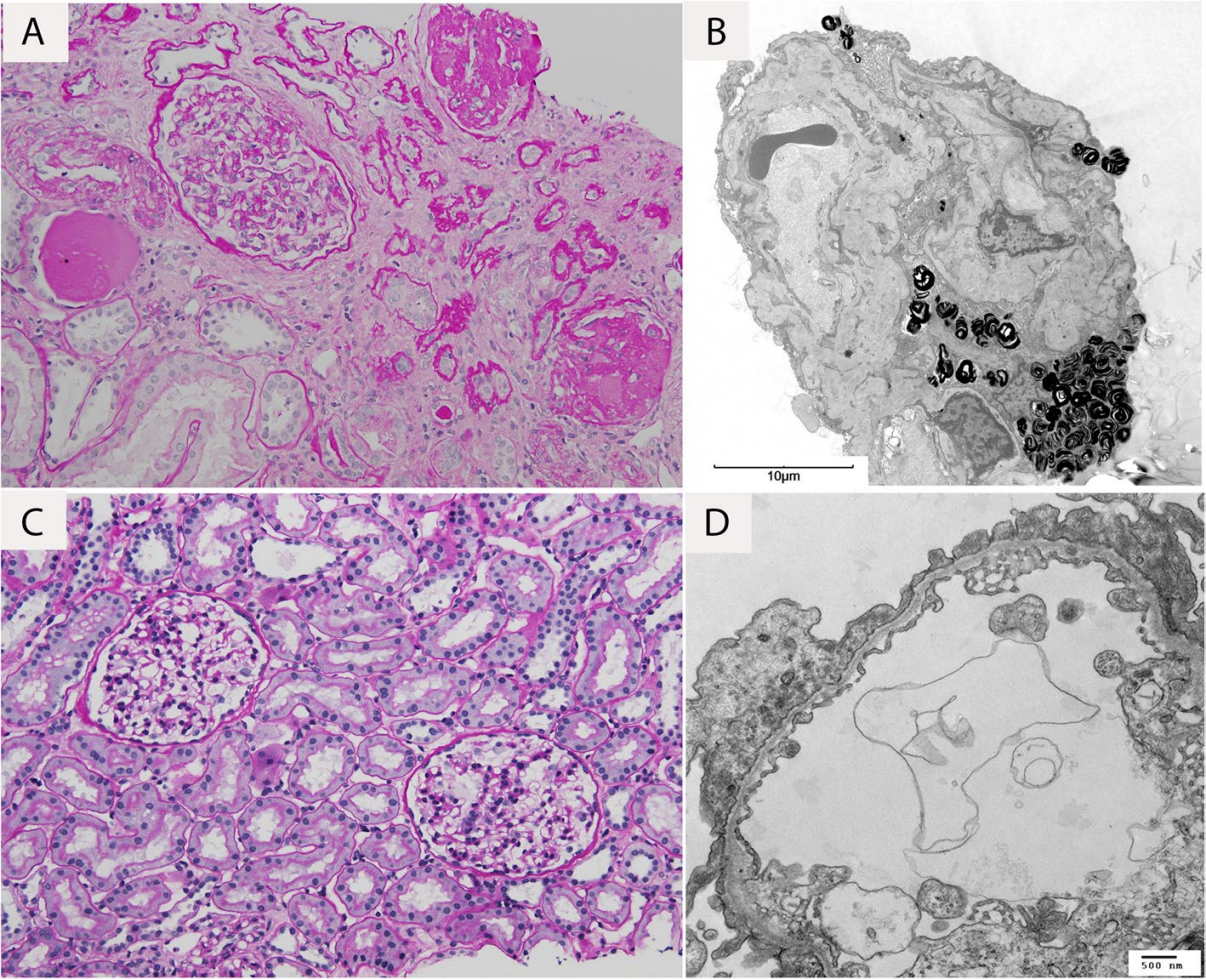
Kidney biopsy findings in patients 1 (**A**, **B**) and 2 (**C**, **D**). (**A**) In patient 1, light microscopy revealed marked non-specific chronic changes, and (**B**) on electron microscopy, there was significant effacement of podocyte foot processes, with attenuation and wrinkling of the basement membranes; numerous lamellar “zebra bodies” were noted in podocyte cytoplasm. (**C**) In patient 2, light microscopy revealed unremarkable parenchyma, while (**D**) electron microscopy was significant for markedly attenuated glomerular basement membranes

**Fig. 2:**
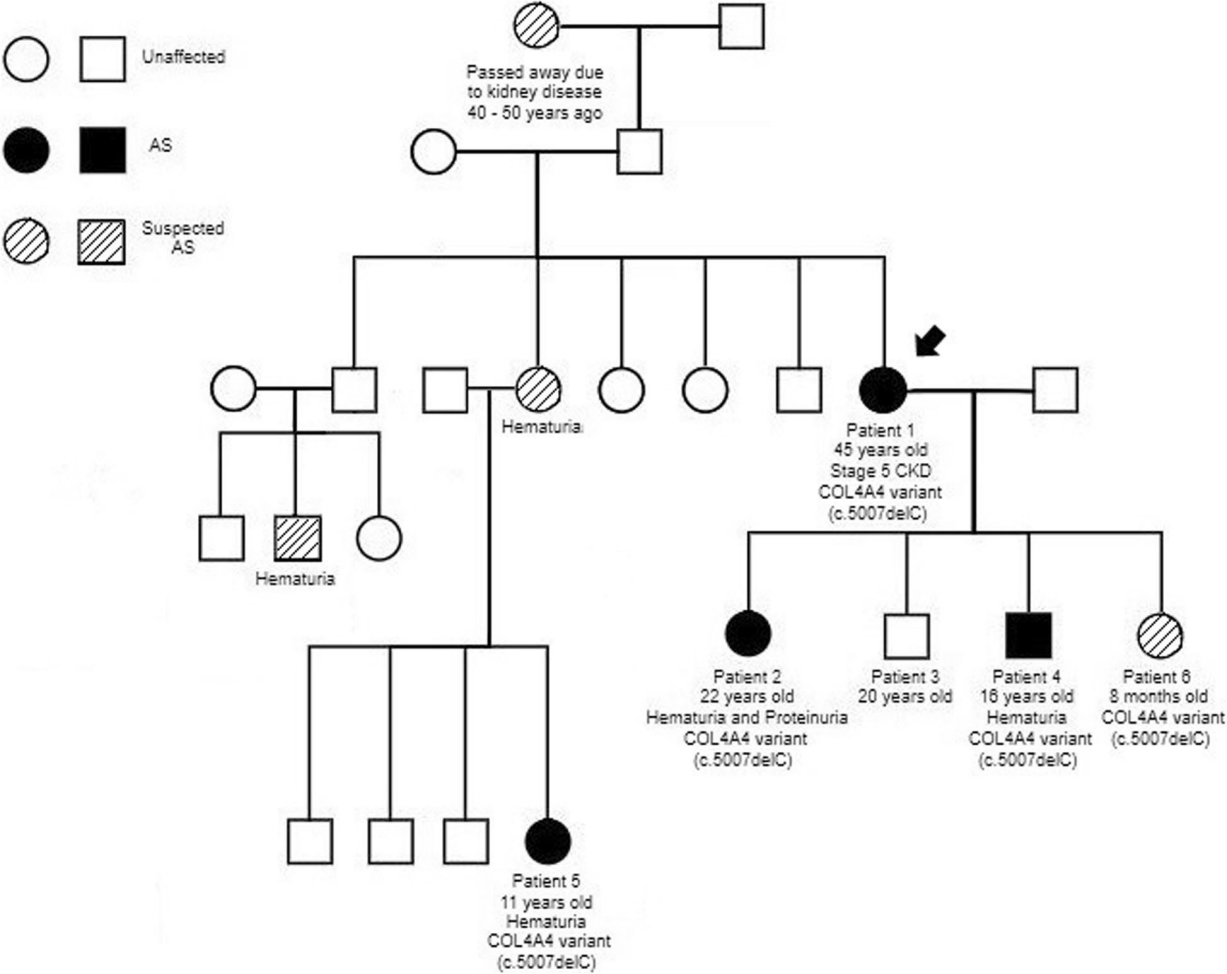
Family pedigree detailed by patient 1. *AS* had genetic and clinical data supportive of diagnosis, *suspected AS* had clinical symptoms without genetic data or genetic data without clinical symptoms, and *unaffected* are healthy individuals

Patient 1 is a 45 year old female who was found to have hematuria at the age of 23, during her first pregnancy and first urinalysis. She had no other significant previous medical history, but she had not seen a physician regularly until her pregnancy due to lack of insurance. During and after her pregnancy, she attended regular and annual appointments with physicians. She first presented to nephrology in 2016, 17 years after her first episode of hematuria, due to right-sided back pain. Her evaluation found microscopic hematuria and proteinuria. She did not receive any additional imaging studies to rule out other causes of her right-sided back pain, but was advised to have a kidney biopsy based on her urinalysis results and elevated creatinine levels. She initially declined. Follow up with her nephrologist showed progressive kidney dysfunction and a serum creatinine of 1.75 mg/dL. She agreed to have a kidney biopsy in 2017, which showed significant chronic changes by light microscopy and podocyte lamellar “zebra bodies” that were suggestive of Fabry disease on electron microscopy (Fig. 1A and B). The findings prompted a referral to medical genetics where further inquiry revealed a significant family history of kidney disease. Her paternal grandmother passed away due to kidney disease 40 –50 years prior, one of her sisters had hearing loss, two of her first cousins had hematuria, her nephew had hematuria, and her niece had hematuria (Fig. 2). Genetic testing for Fabry disease (Invitae, San Francisco, CA, USA) and an alpha-galactosidase enzyme assay (Sema4, Stamford, CT, USA) both came back negative. At that time, the geneticist was also following patient 1’s daughter who was found to have a positive variant of uncertain significance (VUS) in *COL4A4* (patient 2 see below). Due to the previous negative genetic test results, targeted family variant testing was sent for patient 1 for this VUS (GeneDx, Gaithersburg, MD, USA) and she was found to have the same heterozygous nonsense variant of *COL4A4* (c.5007delC (p.Leu1670Ter)) as her daughter (patient 2). Coupling the clinical presentation with the *COL4A4* variant led to a diagnosis of AS for patient 1. Patient 1 was counseled about the risk–benefit of pregnancy, given her diagnosis. However, when she was discovered to be pregnant 3 year later, she chose to carry her pregnancy to term. Patient 1’s kidney disease continued to progress and her kidney function worsened in May 2020 (Table 1), after giving birth to her fourth child (patient 6). Her most recent creatinine is consistent with chronic kidney disease (CKD) stage V and she is not currently on dialysis. She has been treated with Sodium Bicarbonate, Levothyroxine, and Vitamin D3 since October 2019 as well as Ferrous Sulfate since September 2020 and Atorvastatin Calcium since April 2021.

**Table 1:** Clinical information associated with AS in patient 1

Patient 1	Visit 12/2017	Visit 05/2018	Visit 12/2019	Visit 04/2020	Visit 07/2020	Visit 08/2020	Visit 11/2020	Visit 12/2020	Visit 02/2021	Visit 07/2021
Urinary protein/creatinine ratio (P/Cr)	__	__	2.0	__	__	__	3.9	3.4	3.3	1.6
Serum creatinine (mg/dL)	1.85	1.89	2.37	2.43	2.42	2.63	3.07	4.46	4.35	4.6
Estimated glomerular filtration rate (eGFR) (mL/ min/1.73M^2^)	33	32	__	23	24	21	18	11	12	11
Serum total protein (g/dL)	__	7.5	__	8.4	7.2	6.8	__	__	__	__
Albumin (g/dL)	__	3.8	4.0	4.1	__	3.5	__	3.7	4.0	4.1
Blood Pressure (MAP; mmHg)	__	115/73 (87)	__	123/59 (80)	114/56 (75)	105/47 (66)	120/57 (78)	155/75 (102)	150/80 (103)	130/80 (97)
Blood Urinalysis (UA)^a^	Moderate	Moderate	Moderate	__	Large	Moderate	Large	__	__	__

^a^For UA, normal is 0–4 RBCs, trace is 4–6 RBCs, moderate is 6–50 RBCs, and large is > 50 RBCs

Patient 2 is a 22 year old female who was found to have microscopic hematuria on a screening urinalysis at age 5, with worsening proteinuria. She first established care with a pediatric nephrologist in 2015 with isolated hematuria. Initially no biopsy was offered as there was minimal proteinuria, normal renal function, and normotension (Table 2). She had a kidney biopsy performed in January 2018 because of her family history, which was thought at that time to be Fabry disease. The kidney biopsy revealed non-specific changes on light microscopy and thin basement membranes on electron microscopy (Fig. 1C and D). As results were inconsistent with her mother’s biopsy (patient 1) and she had a personal history and family history of hematuria, patient 2 had a genetic test sent for Fabry disease (Invitae, San Francisco, CA, USA) and for thin basement membrane (TBM) disease (GeneDx, Gaithersburg, MD, USA). Her genetic testing results for Fabry disease and TBM were negative, but the TBM results identified a heterozygous nonsense variant of *COL4A4* (c.5007delC (p.Leu1670Ter)) that was classified as a VUS. GeneDx also reported this patient’s variant on ClinVar (ClinVar accession number: SCV001993731.1). As noted above, her mother (patient 1) was found to have the same variant. Patient 2 continues to have hematuria, proteinuria, and intermittent hand and foot pain, but normal kidney function. She has been treated with Enalapril Maleate since December 2015, Vitamin D3 since August 2016, and Gabapentin since January 2019.

**Table 2:** Clinical information associated with AS in patient 2

Patient 2	Visit 04/2015	Visit 11/2015	Visit 12/2015	Visit 02/2016	Visit 08/2016	Visit 08/2017	Visit 11/2017	Visit 01/2018	Visit 01/2019	Visit 07/2020	Visit 04/2021
Urinary protein/creatinine ratio (P/Cr)^b^	0.2	0.6	0.6	0.4	0.1	0.5	0.2	__	0.3	0.3	__
Serum creatinine (mg/dL)	0.50	0.43	0.50	0.43	__	0.57	__	0.55	0.45	0.60	0.61
Serum total protein (g/dL)	7.6	7.7	7.9	7.5	__	7.7	__	7.5	7.4	7.3	7.2
Albumin (g/dL)	4.5	4.7	4.7	4.4	__	4.4	__	4.4	4.4	4.5	4.4
Blood Pressure (MAP; mmHg)	102/61 (75)	98/58 (71)	96/59 (71)	100/58 (72)	91/52 (65)	100/60 (73)	98/58 (71)	100/63 (75)	96/ 61 (73)	105/58 (74)	112/63 (79)
Blood Urinalysis (UA)^a^	Large	Large	Large	Large	Moderate	Large	__	__	Large	Large	Large

^a^For UA, normal is 0–4 RBCs, trace is 4–6 RBCs, moderate is 6–50 RBCs, and large is > 50 RBCs

^b^Patient was initially nonadherent with Enalapril Maleate and that contributed to the fluctuating P/Cr in the beginning, but around late 2017 the patient became more adherent and her P/Cr became more stable

Patient 3 is a 20 year old healthy male. He underwent a Renasight multigene panel for kidney disease (Natera, San Carlos, CA, USA) in August 2021 due to his family history of kidney disease. He was not found to have the same VUS in *COL4A4* that the rest of his family members with AS have. His most recent urinalysis and lab results were normal (Table 3).

**Table 3:** Clinical information associated with AS in patient 3

Patient III	Visit 08/2021
Urinary protein/creatinine ratio (P/Cr)	0.1
Serum creatinine (mg/dL)	0.69
Estimated glomerular filtration rate (eGFR) (mL/ min/1.73M^2^)	137
Serum total protein (g/dL)	7.7
Albumin (g/dL)	5.1
Blood Pressure (MAP; mmHg)	113/70 (84)
Blood Urinalysis (UA)	Negative

Patient 4 is a 16 year old male who was found to have microscopic hematuria at 10 years of age and established care with a pediatric nephrologist in 2015. His most recent urinalysis showed signficant hematuria, and a protein to creatinine ratio of 0.1 mg/mg. His serum creatinine level was 0.83 mg/dL, serum total protein level was 7.3 g/dL, and albumin level was 4.8 g/dL (Table 4). Patient 4 underwent a multigene panel for kidney disease (Invitae, San Francisco, CA, USA) in March 2020 and was found to have the same heterozygous nonsense variant of *COL4A4* (c.5007delC (p.Leu1670Ter)) classified as a VUS, as his mother and sister. In June 2020, Invitae updated the variant associated with AD/AR AS from VUS to pathogenic (Invitae, San Francisco, CA, USA) and reported this patient’s variant on ClinVar (ClinVar accession number: SCV001580980.2). Patient 4 still has microscopic hematuria, but no proteinuria. He has been treated with Vitamin D since March 2020, but is not currently on any other medications.

**Table 4:** Clinical information associated with AS in patient 4

Patient IV	Visit 04/2015	Visit 12/2015	Visit 11/2017	Visit 01/2019	Visit 03/2020	Visit 08/2020	Visit 03/2021
Urinary protein/creatinine ratio (P/Cr)	__	__	0.1	__	0.2	0.1	0.1
Serum creatinine (mg/dL)	__	__	0.59	__	0.47	__	0.83
Serum total protein (g/dL)	__	__	7.8	__	7.7	__	7.3
Albumin (g/dL)	__	__	4.8	__	5.2	__	4.8
Blood Pressure (MAP; mmHg)	__	99/60 (73)	__	107/57 (74)	111/66 (81)	111/55 (74)	114/65 (81)
Blood Urinalysis (UA)^a^	Moderate	Trace—Intact	Trace – Lysed	Trace – Lysed	__	Moderate	Large

^a^For UA, normal is 0–4 RBCs, trace is 4–6 RBCs, moderate is 6–50 RBCs, and large is > 50 RBCs

Patient 5 is an 11 year old female with gross hematuria and was referred to a pediatric nephrologist in 2018. The first urinalysis performed by her pediatric nephrologist after this referral showed large red blood cells and 100 mg/dL protein. She had a protein to creatinine ratio of 0.3 mg/mg, a serum creatinine level of 0.40 mg/dL, and an albumin level of 4.6 g/dL at that time (Table 5). Patient 5 underwent a Renasight multigene panel for kidney disease (Natera, San Carlos, CA, USA) in June 2021 because of her aunt’s recent diagnosis of AS (patient 1), her cousins’ clinical symptoms (patients 2 and 4), and her mother’s history of having microscopic hematuria. Patient 5 was found to have the same heterozygous nonsense variant of *COL4A4* (c.5007delC (p.Leu1670Ter)) classified as a VUS, as her aunt and cousins. Patient 5 still has microscopic hematuria, has trace protein in her urine, and has not had a kidney biopsy. Her other laboratory results are normal and she is not currently on any medications.

**Table 5:** Clinical information associated with AS in patient 5

Patient V	Visit 9/2017	Visit 2/2018	Visit 10/2018	Visit 1/2019	Visit 06/2021	Visit 08/2021
Urinary protein/creatinine ratio (P/Cr)	__	0.3	__	0.2	0.2	__
Serum creatinine (mg/dL)	0.47	0.40	0.41	__	0.46	0.43
Serum total protein (g/dL)	__	__	__	__	6.7	__
Albumin (g/dL)	4.4	4.6	4.9	__	4.7	4.5
Blood Pressure (MAP; mmHg)	__	__	__	__	120/70 (87)	__
Blood Urinalysis (UA)^a^	Moderate	Large	Large	Large	Moderate	__

^a^For UA, normal is 0–4 RBCs, trace is 4–6 RBCs, moderate is 6–50 RBCs, and large is > 50 RBCs

Patient 6 is an 8 month old female who underwent a Renasight multigene panel for kidney disease (Natera, San Carlos, CA, USA) in December 2020 because of her mother’s (patient 1) concern of AS. It was also found that patient 6 had the same heterozygous nonsense variant of *COL4A4* (c.5007delC (p.Leu1670Ter)) classified as a VUS, as her mother (patient 1), affected siblings (patients 2 and 4), and maternal 1^st^ cousin (patient 5). In August 2021, Natera updated the *COL4A4* variant from VUS to likely pathogenic (Natera, San Carlos, CA, USA). This was due to the segregation of this variant with AS in the family and the clinical information provided by affected family members. Her most recent serum creatinine level was 0.33 mg/dL and her blood pressure was 99/61 on December 2020 (Table 6). She is not currently taking any medications. More information regarding the *COL4A4* variant can also be seen in Table 7.

**Table 6:** Clinical information associated with AS in patient 6

Patient VI	Visit 12/2020
Urinary protein/creatinine ratio (P/Cr)	__
Serum creatinine (mg/dL)	0.33
Estimated glomerular filtration rate (eGFR) (mL/ min/1.73M^2^)	__
Serum total protein (g/dL)	__
Albumin level (g/dL)	__
Blood Pressure (MAP; mmHg)	99/61 (74)

**Table 7:** Information about the *COL4A4* variant

Features	*COL4A4* Variant
Type of Variant	Nonsense
RefSeq	NM_000092.5
HGVS Coding	c.5007del
HGVS Protein	p.Leu1670Ter
Cytogenetic Location	2q36.3
ExAC Frequency	0
gnomAD Control Frequency	0
PhyloP100way Score	8.032
ClinVar Invitae Accession Number	SCV001580980.2
ClinVar GeneDx Accession Number	SCV001993731.1
Natera ACMG Criteria Scores	PVS1_strong, PM2_supporting, PP1_supporting
Invitae Sherloc System Criteria and Point Values	•LOF variant and LOF is a known mechanism of disease in *COL4A4* with absence in the ExAC population (+ 2.5 points for Sherloc scoring system and similar to Natera’s PVS1 and PM2) •Discovery of another patient in their laboratory that had a pathogenic variant upstream this case’s variant (+ 2.5 points for Sherloc scoring system and did not directly correlate with evidence from the ACMG criteria)
GeneDx ACMG Criteria Score	PVS1 (strength decreased to strong)

## Discussion and Conclusions

In this report, a *COL4A4* heterozygous variant, not yet discussed in detail in the literature, was shown to segregate with features of AS in two generations of a single family. The reported variant, c.5007delC (p.Leu1670Ter), is classified by consensus as likely pathogenic among different clinical genetic testing laboratories and is a nonsense variant in the *COL4A4* gene. These findings were disclosed to all patients in the case report. In addition, the pattern of segregation with disease in affected family members that only had one copy of this variant, suggests an AD mode of inheritance. The classification of the reported variant changed over time based on additional clinical information regarding phenotype, family history, and the role of the variant in protein translation. This case highlights the challenges that arise in the current era of genetic medicine. Clinicians should ensure they obtain a thorough family history and collaborate with geneticists and genetic counselors to verify that all variants associated with a disease of interest, not just known pathogenic variants, are evaluated. This case also demonstrates how genetic testing can lead to non-invasive, early diagnosis and improve monitoring, prevention, and/or mitigation of disease progression.

Next-generation sequencing and similar technologies allow researchers to investigate the significance of disease-associated variants cost-effectively and quickly for clinical diagnoses [20–25]. Consistency between the significance of disease-associated variants is necessary [16, 17]. Thus in 2015, the ACMG developed a framework for variant interpretation and determination of pathogenicity. Each variant classification is assigned a direction, benign or pathogenic, and the evidence criteria for classification is based on a level of strength: stand‐alone (A), very strong (VS), strong (S), moderate (M), or supporting (PP) [15]. Despite these guidelines, nuanced classifications of variants and internal data can lead to different classifications of the same variant by separate genetic testing laboratories, such as Natera, Invitae, and GeneDx in this case report.

One of the salient points of this case is that the variant’s classification changed overtime. Initially, Natera (Natera, San Carlos, CA, USA) classified the variant as a VUS using a modified version of the ACMG criteria. The variant in this case results in the conversion of leucine to a premature termination at nucleotide 198 of 4875 of exon 48. This nonsense variant, which is located in the last exon, is not anticipated to result in nonsense mediated decay and therefore interpretation of such variants is always made with caution (PVS1_strong) [15, 26, 27]. In addition to the low frequency of the variant in gnomAD (PM2_supporting), the newly provided evidence from the co-segregation data (PP1_supporting) was sufficient to upgrade its classification to likely pathogenic [20]. Invitae (Invitae, San Francisco, CA, USA) used the Sherloc scoring system, which cannot be directly compared with the ACMG framework for variant classification. They used evidence that it is a loss of function (LOF) variant and that LOF is a known mechanism of disease in *COL4A4*, similar to Natera’s PVS1, for their original VUS classification [28–30]. Their classification was upgraded from VUS to pathogenic due to the discovery of another patient in their laboratory that had a pathogenic variant upstream this case’s variant. The upstream variant is a nonsense variant that causes protein function loss, which did not directly correlate with evidence from the ACMG criteria. In their upgrade, they also included the variant’s absence in the population according to ExAC (ClinVar accession number: SCV001580980.2), which was similar to Natera’s PM2. GeneDx only incorporated the information that this was a nonsense variant in a gene where LOF is a known mechanism of disease and that it was predicted to disrupt the last 21–23 amino acids of the protein (PVS1). However, because the variant’s location was so close to the C-terminus or 3’ end of the gene, the evidence’s strength was decreased to strong evidence. This led GeneDx to classify this variant as a VUS and as of the date of this publication, the variant remains a VUS (ClinVar accession number: SCV001993731.1). Although the classification varied among these different clinical genetic testing laboratories, the authors agree that by consensus this variant is likely pathogenic.

According to the Online Mendelian Inheritance in Man (OMIM) data, pathogenic variants in *COL4A4* are associated with ARAS or ADAS, which can be difficult to distinguish when observing the early clinical symptoms of AS [31]. However, the *COL4A4* heterozygous variant described in this case appears to have an AD mode of inheritance. In addition, patient 3, who was 20 years old and healthy, lacked the variant.

Phenotypically, age-related penetrance and the spectrum of phenotypes of a single variant are important to take into consideration when evaluating *COL4A4* ADAS [11, 12]. Kidney biopsies of patients with ADAS demonstrate a predominance of thickening and thinning of the GBM, and less commonly, wrinkling of the GBM and foot process effacement [11]. The histology observed in our patients have similarities with the kidney biopsy reports of patients heterozygous for a pathogenic variant in *COL4A4*. Patient 1’s biopsy was atypical regarding the numerous lamellar “zebra bodies”. However, patient 1’s biopsy had significant effacement of podocyte foot processes with wrinkling of the basement membranes and patient 2’s biopsy showed alternate thinning and thickening of the GBM without lamellations, which were consistent with kidney biopsy findings from *COL4A4* heterozygotes.

The mean age of onset of kidney failure in ADAS is 52.8 years. However, the type of variant impacts the timing of kidney failure. Patients who have variants that lead to a premature termination of translation develop kidney failure at 47.1 years compared to 55.2 years in patients with missense variants [11]. Most of the patients in this case report seem to have a disease progression consistent with patients with ADAS, except for patient 1. As compared to reports of mild disease, patient 1’s disease progression was very rapid. There are a couple of factors that likely accelerated patient 1’s disease course. There was a delay in the diagnosis and monitoring of this patient because she did not have routine follow up and care with a nephrologist. Second, she had multiple pregnancies, which in turn can accelerate CKD progression [23]. Aside from her disease course, patient 1 exhibited the most common symptoms associated with AS, hematuria and proteinuria. The other affected patients in this family also display common AS symptoms. The older affected children (patients 2 and 4) have more clinical features, due to age-related penetrance.

Based on the co-segregation and findings, it is very convincing that this variant is contributing to pathogenicity. However, it should be noted that there were some limitations in this case report. The classifications from all these genetic testing laboratories indicated PVS1 as evidence for pathogenicity. However, more research as to the exact impact of the variant on the translated protein is still needed. In addition, patients only received gene panel testing, and not whole exome or whole genome sequencing. Thus, we cannot definitively conclude whether another variant in an untested gene may be contributing to this family’s phenotype. Patient 2 experienced neuropathic pain, not typically associated with AS, and the clinical team believes that this is caused by another clinical condition. Patient 1’s biopsy displayed numerous lamellar “zebra bodies”, which was also atypical. The clinical team is unsure as to what caused this.

Defining the pathogenicity of this *COL4A4* variant has several clinically meaningful implications*.* All the proband’s children, except for patient 2, were spared an invasive biopsy. Follow up and monitoring by a nephrologist was started early on, prior to onset of several clinical symptoms. Patient 2 was treated with an angiotensin-converting enzyme inhibitor early on and counseled about the risk–benefit of pregnancy. Patients and parents were clinically informed about nephroprotective measures, such as avoiding NSAIDs, nephrotoxins, and maintaining a healthy diet.

In conclusion, we are the first to describe a family with this c.5007delC (p.Leu1670Ter) *COL4A4* variant in detail in the literature. This is a variant classified as likely pathogenic by consensus and has an AD mode of inheritance. Nephrologists should recognize the benefit of collaborating with genetic laboratories and share the relevant clinical and family history of the patient to appropriately classify variants and determine pathogenicity. In turn, it would benefit genetic testing laboratories to regularly update their data and find centralized ways, in addition to ClinVar and ClinGen, to share their molecular data. Proper diagnosis, consistent classification of variants, and early monitoring can be beneficial for the treatment of patients with AS.

## Data Availability

Records and data pertaining to this case are in these patients’ secure medical records but can be made available with appropriate HIPAA compliance from the corresponding author on reasonable request.

## Abbreviations

AS: Alport syndrome
CKD: Chronic kidney disease
GBM: Glomerular basement membrane
ACMG: American College of Medical Genetics and Genomics
VUS: Variant of uncertain significance
AD: Autosomal dominant
AR: Autosomal recessive
TBM: Thin basement membrane
TBMN: Thin basement membrane nephropathy
ADAS: Autosomal dominant Alport syndrome
ARAS: Autosomal recessive Alport syndrome
OMIM: Online Mendelian Inheritance in Man
LOF: Loss of function
A: Stand‐alone
VS: Very strong
S: Strong
M: Moderate
PP: Supporting
